# Determinants of Malnutrition and its associated factors among pregnant and lactating women under armed conflict areas in North Gondar Zone, Northwest Ethiopia: a community-based study

**DOI:** 10.1186/s40795-023-00758-1

**Published:** 2023-09-04

**Authors:** Aysheshim Kassahun Belew, Tadesse Awoke, Kassahun Alemu Gelaye, Asmamaw Atnafu, Tadesse Guadu, Telake Azale, Mezigebu Yitayal, Yohannes Awoke Assefa, Rediet Getachew, Tadele Amare, Sewbesew Yitayih, Kegnie Shitu, Demeke Demilew, Endalkachew Dellie, Andualem Yalew Aschalew, Biruk Fanta, Netsanet Worku, Ermias Solomon Yalew, Yohannes Abich, Getachew Azeze, Chanyalew Worku, Alemu Kassaw Kibret, Tsegaye G/Medhin, Melkamu Tamir Hunegnaw, Endalamaw Salelew, Goshu Nenko, Hailab Fekadu, Ayenew Molla

**Affiliations:** 1 Department of Human Nutrition, Institute of Public Health, College of Medicine and Health Sciences, University of Gondar, Gondar, Ethiopia; 2 Department of Epidemiology and Biostatistics, Institute of Public Health, College of Medicine and Health Sciences, University of Gondar, Gondar, Ethiopia; 3 Department of Health system and Policy, Institute of Public Health, College of Medicine and Health Sciences, University of Gondar, Gondar, Ethiopia; 4 Department of Environmental and Occupational Health and Safety, Institute of Public Health, College of Medicine and Health Sciences, University of Gondar, Gondar, Ethiopia; 5 Department of Health Promotion and Health Behavior, Institute of Public Health, College of Medicine and Health Sciences, University of Gondar, Gondar, Ethiopia; 6 Department of occupational therapy, School of Medicine, College of Medicine and Health Sciences, University of Gondar, Gondar, Ethiopia; 7 Department of physiatriy, School of Medicine, College of Medicine and Health Sciences, University of Gondar, Gondar, Ethiopia; 8 Department of Physiotherapy, School of Medicine, College of Medicine and Health Sciences, University of Gondar, Gondar, Ethiopia; 9 Department of Medical Nursing, School of Nursing, College of Medicine and Health Sciences, University of Gondar, Gondar, Ethiopia

**Keywords:** Malnutrition, Pregnant and lactating mother, North Gondar Zone, War, Ethiopia

## Abstract

**Introduction:**

Maternal malnutrition remains a major public health problem, particularly in low and middle-income countries and war-affected areas like Ethiopia. Malnourished pregnant and lactating women with low nutrient stores have babies with poor mental and physical development, increasing the risk of poor birth outcomes. Despite the fact that the majority of Ethiopian mothers are malnourished, there is little evidence in war-affected areas. Therefore, the objective of this study was to assess the prevalence of undernutrition and associated factors among pregnant and lactating mothers in the war affected area of North Gondar Zone, northwest Ethiopia.

**Methods:**

A community-based cross-sectional study was conducted from April 10 to May 25, 2022. A multistage random sampling technique was used to select 1560 pregnant and lactating mothers. MUAC was to ascertain the outcome variable. Data was entered and analyzed by using EPI INFO version 3.5.3 and SPSS version 24, respectively. A multivariable logistic regression analysis was employed to identify the factors associated with acute malnutrition. An adjusted odds ratio (AOR) with a 95% confidence interval was used to show the strength of the association, while a P-value of 0.05 was used to declare the significance of the association.

**Results:**

The prevalence of acute malnutrition among pregnant and lactating women was 34.3% at the 95% CI (31.9–36.8). The age of the mothers (AOR = 0.73; 95% CI: 0.54, 0.99), family size 6–8 (AOR = 1.21; 95% CI: 1.03, 1.82), and greater than or equal to 9 family sizes (AOR = 0.44; 95% CI: 0.19, 0.97), were significantly associated with acute malnutrition.

**Conclusions:**

In the current study, the prevalence of acute malnutrition among pregnant and lactating mothers is high in the study area. Mother’s age and family size were factors associated with acute malnutrition in war-affected areas. As a result, mothers with large families will require special assistance to reduce the impact of malnutrition.

## Introduction

Violent conflicts have become more complex and protracted, involving more non-state groups and regional and international actors. As a result, more than a quarter of the world’s population lives in war-affected areas [1], resulting in an influx of acute malnutrition and a high prevalence of micronutrient deficiencies among the most vulnerable in the humanitarian context, particularly under five children and pregnant and lactating mothers due to prolonged starvation and disease [2–4].

According to the United Nations (UN) 2022 report, over 828 million people worldwide are malnourished, with 349 million affected by food insecurity as a result of the combined effects of conflict, COVID, the climate crisis, and rising costs, increasing the prevalence of acute malnutrition among pregnant and lactating mothers in low and middle-income countries [5, 6]. Armed conflict is a major contributor to 60% of people experiencing acute food insecurity [7]. In the global report on food crises by 2022, 140 million people in Africa were suffering from acute food insecurity [8], and the 2023 report indicated that more than 22.6 million Ethiopians require food due to drought, conflict, and an increase in food prices [9], primarily in conflict-affected areas of the Afar, Amhara, and Tigray regions [10].

Reports from 2022 show that 1.8 million pregnant and lactating women need food assistance in the war affected area [10].

Pregnant and lactating women and war are linked due to their higher nutritional needs and the detrimental effects of poor nutrition on the health of the mothers and their children. Under the normal situation, about 22% of women are chronically energy deficient; 28% of pregnant and lactating women are anaemic [11]; 37.9% are vitamin A deficient [12]; and 77.6% are iodine deficient [13]. Similarly, one third of all women of reproductive age have anaemia, and millions of women are still underweight, and the effect is high in armed conflict areas [14].

Preterm labour, perinatal mortality, and low birth weight are all associated with poor nutritional status among pregnant women [15–17]. In addition, poor growth and development raises the risk of contracting infectious diseases such as malaria, increasing morbidity and mortality in children, and developing non communicable diseases such as type 2 diabetes and heart conditions in adulthood [18–20]. Furthermore, poor malnutrition among lactating women in armed conflict areas increased low levels of vitamins such as vitamin B, A, and essential fatty acids [21, 22], which translated into low breast milk concentrations, leaving infants more vulnerable to inadequate consumption, potentially with negative consequences for their cognitive development [18, 22].

During a crisis, malnutrition among pregnant and lactating women is associated with a variety of factors, including food supply shortages, fortified foods that may cause micronutrient deficiencies, increased psychological problems, and gender violence, which increases the risk of nutritional vulnerability among pregnant and lactating mothers [4, 23, 24]. Poverty, food insecurity, gender discriminatory food allocation, food avoidance, and a lack of access to adequate health services all contribute to poor nutritional status in women [25].

With the collaboration of various governmental and non-governmental organizations, global and national nutrition clusters are working to provide lifesaving nutrition activities in war and other affected areas to the most vulnerable groups, such as infants and children and pregnant and lactating mothers, with specific nutrition needs such as screening, counseling, and supplementation [26]. In addition, routine nutrition screening is implemented in all regions of the country every three months, but malnutrition among PLW remains a major public health problem in Ethiopia [27, 28]. Therefore, regular monitoring of PLW nutritional status aids in the provision of baseline evidence as well as the monitoring and evaluation of current intervention programs in areas of persistent armed conflict. However, no other similar study in the war affected areas is known about the impact of armed conflict on malnutrition in Ethiopia, including the study area. As a result, the purpose of this study was to determine the prevalence and associated factors of acute malnutrition among pregnant and lactating mothers in war-affected areas of the North Gondar Zone in northwest Ethiopia.

## Methods

### Study setting and design

A community based cross-sectional study was conducted from April 10 to May 25, 2022, in the North Gondar Zone, northwest Ethiopia. North Gondar Zone is one of the Amhara region’s most severely affected areas by war. The zone has eight districts and two city administrations, with five district hospitals, 83 health centres, and 271 health posts. During the time of data collection, three hospitals, 68 health centers, and 211 health posts were functional. Based on the Central Statistics Agency population projection for 2022, a total of 102,845 people live in the zone. The war affected site in North Gondar Zone has a total of 23 Kebeles (Ethiopia’s smallest administrative unit). In addition, the area has a total of three internally displaced person (IDP) sites, such as Dabat, Debark, and Zarima, with a total population of 9887 (5578 females and 4309 males). Furthermore, the host community has a total population of 126,237 people.

### Sample size and sampling procedure

All pregnant and lactating mothers living in war-affected areas in the north Gondar zone were the source population for this study. The sample size was calculated using a single proportion formula by considering the following assumptions: a 24% prevalence of malnutrition among pregnant and lactating women under humanitarian settings in Ethiopia [28], a 95% confidence level, and a 3% degree of precision. Finally, the sample size of 1635 was obtained by considering the 5% non-response rate and the design effect of two.

A multistage random sampling technique was used. Three districts were identified from the zone as they were affected by the war. Study participants were proportionally allocated to the selected districts. A total of 12 kebeles were selected randomly from the districts. Finally, using simple random sampling techniques, study subjects were randomly selected from the selected kebeles.

### Data collection tool and procedures

A structured face-to-face interview questionnaire was used to collect data. The tool was developed by reviewing similar studies [29–31]. To ensure consistency, the questionnaire was developed in English, translated into Amharic, and then translated back into English by an English language and public health expert. A pretest was done on 5% of the sample outside of the study area. Data collectors and supervisors were trained for two days about methods of interview and anthropometric measurements. A total of 16 public health professionals as data collectors and 4 MPH/MSc nutrition experts as supervisors were recruited for the study. During the data collection, close supervision was done by the principal investigator, supervisors, and research team members.

### Variable measurements

In resource-poor settings and armed conflict situations, where pregnant and lactating women tend to have smaller amounts of subcutaneous fat, changes in the mid-upper-arm circumference (MUAC) are more likely to reflect changes in muscle mass. Therefore, MUAC measurements can be useful as an indicator of protein-energy malnutrition or starvation, particularly in a humanitarian context [32, 33]. In addition, the SPHERE Guidelines also recommend the use of MUAC for PLW screening and the entry of PLW into feeding programs. However, MUAC cutoff points vary by country, typically ranging from 21 to 23 cm, and values of 21 cm could be used to identify and target PLW at risk of growth retardation [34]. In addition, the Ethiopian Emergency Nutrition Coordination Unit (ENCU) recommends using MUAC less than 21 cm as a cut point to classify acute malnutrition in emergency settings [35].

MUAC was measured to the nearest 0.1 cm at the mid-point between the tips of the shoulder and elbow of the left arm using non-elastic and non-stretchable MUAC tapes. The subject stood up on the basal part of the device with his feet together. Bend the left arm at a 90-degree angle, straighten the arm, and wrap the tape around the arm at the midpoint. Place the tape through the window, and correct the tape tension, and read the measurement in centimeters (cm) in the window where the arrows point inward. The measurer records the measurement to the nearest 0.1 cm and records the colour. Considering the standardized criteria, a MUAC value of less than 21 cm was categorized as acute malnutrition, whereas those with a MUAC greater than or equal to 21 cm were classified as normal [36].

### Data processing and analysis

All questionnaires that were returned were manually reviewed for completeness and consistency of responses. Then, the collected data were entered into EPI-INFO version 3.5.3 and exported to SPSS version 20 for further analysis. Descriptive statistics, such as figures, tables, and frequencies, were used to summarize variables. Both bivariable and multivariable binary logistic regression analyses were used to identify factors associated with acute malnutrition. Variables with a p-value less than 0.2 in the bivariable analysis were fitted into the multivariable logistic regression analysis. Both the Crude Odds Ratio (COR) and Adjusted Odds Ratio (AOR) with the corresponding 95% confidence interval were calculated to show the strength of the association. Finally, in the multivariable analysis, variables with a P-value less than 0.05 were considered statistically significant.

## Results

### Sociodemographic characteristics of the participants

In the current study, a total of 1560 pregnant and lactating mothers, with a 95.4% response rate, participated. The median age of the respondents was 30 (inter-quartile range, IQR; 26–36) years. More than one-third 552 (35.4%) of the study participants were between the ages of 31 and 40. The majority of 1330 (85.3%) of the study participants were married at the time of data collection. Almost three-quarters of the 1112 study participants (71.3%) were unable to read and write, and nearly all 1503 (96.3%) of the pregnant and lactating mothers were Orthodox. In terms of occupation, the majority of study participants 1487 (85.3%) were housewives, and 1288 (96.8%) of study participants’ husbands were farmers. Two-thirds 970 (62.2%) of pregnant and lactating mothers have a family size of three to five children (Table 1).

**Table 1:** Sociodemographic characteristics of pregnant and lactating mothers in the war affected area, North Gondar Zone, Northwest Ethiopia, 2022

Variables	Frequency	Percentage (%)
**Age of the mothers (Years)**		
18–25	361	23.1
26–30	460	29.5
31–40	552	35.4
41–45	187	12.0
**Religion**		
Orthodox	1503	96.3
Muslim	57	3.7
**Marital status**		
Married	1330	85.3
Unmarried	230	14.7
**Maternal education status**		
Unable to read and write	1112	71.3
Able to read and write	273	17.5
Primary education	163	10.4
Secondary education and above	12	0.8
**Husband education status(n = 1330)**		
Unable to read and write	817	61.4
Able to read and write	381	28.7
Primary education and above	132	9.9
**Maternal occupation**		
Housewife	1487	85.3
Daily laborer	51	3.3
Student	16	1.0
Employee	6	0.4
**Husband occupation(n = 1330)**		
Farmer	1288	96.8
Employee	17	1.3
Daily laborer	25	1.9
**Family size**		
<=2	190	12.2
3–5	970	62.2
6–8	370	23.7
>=9	30	1.9

### Health service utilization and food source

Nearly two-thirds 924(59.2%) of the study participants were lactating mothers. The majority 523(82.2%) of the mothers were not receiving all forms of antenatal care (ANC) for their current pregnancy. The majority 745(80.6%) of study participants were not receiving postnatal care (PNC) services for a recent child. More than a quarter 458 (29.4%) of pregnant and lactating women received only 1–2 meals per day. Food aid provided food for more than two-thirds 1096 (70.3%) of pregnant and lactating mothers (Table 2).

**Table 2:** Pregnant and lactating mothers’ use of health services and food sources in a war-affected area, North Gondar Zone, Northwest Ethiopia, 2022

Variables	Frequency	Percentage
**Physiological State/condition**		
Pregnant	636	40.8
Lactating	924	59.2
**ANC service(n = 636)**		
No	523	82.2
Yes	113	17.8
**PNC visits for recent child(n = 924)**		
No	745	80.6
Yes	179	19.4
**Number of meals per day**		
1–2 times	458	29.4
3 times	985	63.1
>=4 times	117	7.5
**Family food Source**		
Food aid	1096	70.3
Purchasing	357	22.9
Cultivation/production	107	6.9

NB: ANC = 636 was considering only pregnant mothers at the time of data collection and PNC = also considered only from lactating mothers

### Prevalence of undernutrition among pregnant and lactating women

The MUAC measurements of 1560 pregnant and lactating mothers were taken, with a mean of 22.9 cm and a standard deviation of 2.89 cm. The overall prevalence of acute malnutrition among pregnant and lactating mothers in war-affected areas was 34.3% at the 95% CI (31.9–36.8).

### Factors associated with undernutrition

The results of the multivariable logistic regression revealed that the mother’s age and family size were significantly associated with acute malnutrition.

Mothers’ age 26 to 30 years old was 27% times (AOR = 0.73; 95% CI: 0.54, 0.99) less likely to be affected by acute malnutrition as compared to those aged 18 to 25 years old. Pregnant and lactating mothers who had a family size of 6 to 8 were 1.21 (AOR = 1.21, 95% CI: 1.03, 1.82) more likely to be affected by acute malnutrition as compared to those with less than or equal to two family sizes. In addition, pregnant and lactating mothers who had greater than or equal to 9 family sizes were 56% (AOR = 0.44, 95% CI: 0.19, 0.97) less likely to be affected by acute malnutrition as compared to those with less than or equal to 2 family sizes (Table 3).

**Table 3:** Bivariable and multivariable logistic regression output showing that factors associated with undernutrition among pregnant and lactating mothers in war-affected areas, Central Gondar Zone, Northwest Ethiopia, 2022

Variables	Nutritional status		Crude Odds Ratio with 95% C)	Adjusted Odds Ratio with 95% CI
	Malnourished(< 21 cm)	Normal( > = 21 cm)		
**Age of the mothers**				
18–25	111(30.7%)	250(69.3%)	1	1
26–30	169(36.7%)	291(63.3%)	0.77(0.57,1.03)	0.73(0.54, 0.99)*
31–40	193(35%)	359(65%)	0.83(0.62,1.09)	0.78(0.58, 1.05)
41–45	62(33.2%)	125(66.8%)	0.89(0.61, 1.31)	0.86(0.57, 1.28)
**Marital Status**				
Married	442(33.4%)	880(66.6%)	1	1
Unmarried	93(39.1%)	145(60.9%)	0.89(0.69, 1.26)	0.82(0.61, 1.14)
**Educational status of the mother**				
Unable to read and write	377(34.2%)	726(65.8%)	1.95(0.63, 6.09)	1.88(0.59, 5.97)
Able to read and write	99(35.1%)	183(64.9%)	1.90(0.60, 6.07)	1.73(0.54, 5.60)
Primary Education	53(32.5%)	110(67.5%)	2.14(0.66, 6.94)	2.01(0.61, 6.61)
Secondary education and above	6(50%)	6(50%)	1	1
**Family size**				
<=2	72(37.9%)	118(62.1%)		
3–5	323(33.3%)	647(66.7%)	1.22(0.89, 1.69)	1.20(0.89, 1.77)
6–8	123(33.2%)	247(66.8%)	1.23(0.85, 1.76)	1.21(1.03, 1.82)*
>=9	17(56.7%)	13(43.3%)	0.47(0.21, 1.02)	0.44(0.19, 0.97)*
**Numbers of meals per day(times)**				
<=2	157(34.3%)	301(65.7%)	1.33(0.88, 2.02)	1.32(0.89, 2.11)
3	330(33.5%)	655(66.5%)	1.38(0.93, 2.04)	1.36(0.93, 2.05)
4–6	48(41%)	69(59%)	1	1
**Physiological state**				
Lactating	304(32.9%)	620(67.1%)	1.16(0.94, 1.44)	0.87(0.70, 1.08)
Pregnant	231(36.3%)	405(63.7%)	1	1

* indicate significant at p value less than 0.05 in multivariable logistic analysis

## Discussion

The current study is a prelude to the testimony on the prevalence and determinants of malnutrition among PLW in Ethiopia’s war-affected areas. According to this study, more than one-third (34.3%) of pregnant and lactating women were severely malnourished. The finding is higher than that of the study done in the Rayitu district humanitarian area in Ethiopia [28]. According to reports from Rayitu district, there are no assets that have not been damaged or robbed by militants, whereas in the current study areas, almost all fixed assets have been damaged and looted during the war. Furthermore, during the war, there was population displacement, which exacerbated the effects of acute malnutrition among the most vulnerable segments of the population, such as pregnant and lactating mothers. Furthermore, since the previous study report was done in a drought prone and food-insecure area, there might be regular and periodic food aid supplementation by the humanitarian agencies as compared to when people living in war-affected areas.

Mothers’ age of 26 to 30 years old was 27% less likely to be affected by acute malnutrition as compared to those aged 18 to 25 years old. This could be because pregnant and lactating women, even at a young age, may be aware of the benefits of good nutrition for themselves and their children. When compared to women over the age of 26, the number of parities is lower when they are young and may not affect their food intake, either by preparing over a long period of time or by decreasing their intake of portion size as a result of war-driven food insecurity.

Pregnant and lactating mothers who had a family size of 6 to 8 were 1.21 times more likely to be affected by acute malnutrition as compared to those with less than or equal to two family sizes. The possible reason might be due to the fact that having a large family will take a long time to prepare food and affect the regular meal schedule of pregnant and lactating women. An outsized family member needs an adequate amount of food, but as a result of the war, there might be a small amount of food for each family member, which reduced their food intake and increased the risk of malnutrition [27]. The food aid program focuses on severe acute malnutrition and moderate malnutrition rather than delivering general food rations. Furthermore, overcrowding is positively associated with malnutrition; a large family size causes overcrowding and determines a higher risk of disease presence, primarily diarrhoea and infectious diseases that cause weight and height detriments in pregnant and lactating mothers. Mothers may take food after all the family members have eaten, which causes a low intake of their food for their infants and unborn neonates. On the contrary, pregnant and lactating mothers who had greater than or equal to 9 family sizes were 56% less likely to be affected by acute malnutrition as compared to those with less than or equal to 2 family sizes. The possible reason might be having a higher probability of task sharing to reduce the workload of the mother because work load is a possible cause of malnutrition due to high energy expenditure [37].

### Strengthen and limitation

as strength, the research was conducted in areas where no other similar studies had been conducted. The strength is the identification and operation in an emergency context where little similar research was done. The study limitations overlook some variables, like food security, dietary and feeding practices, and disease states among pregnant and lactating women. In addition, the nature of the study design makes it difficult to explain causality among variables.

## Conclusion

In the current study, the prevalence of acute malnutrition among pregnant and lactating mothers is high in the study area. The mother’s age and family size were factors associated with acute malnutrition in war-affected areas. As a result, mothers with large families will require special assistance to reduce the impact of malnutrition.

## Data Availability

Data will be available upon request from the corresponding authors.

## Abbreviations

ANC: Antenatal Care
AOR: Adjusted Odds Ratio
CI: Confidence Interval
CM: Centimeter
COR: Crud Odds Ratio
ENCU: Ethiopian Emergency Nutrition Coordination Unit
IQR: Inter-Quartile Range
MUAC: Middle Upper Arm Circumference
PLW: Pregnant and Lactating Mother
PNC: Postnatal Care
SPSS: Statistical Package for Social Sciences
