# Correction: Determinants of Malnutrition and its associated factors among pregnant and lactating women under armed conflict areas in North Gondar Zone, Northwest Ethiopia: a community-based study

**DOI:** 10.1186/s40795-024-00855-9

**Published:** 2024-03-06

**Authors:** Aysheshim Kassahun Belew, Tadesse Awoke, Kassahun Alemu Gelaye, Asmamaw Atnafu, Tadesse Guadu, Telake Azale, Mezigebu Yitayal, Yohannes Awoke Assefa, Rediet Getachew, Tadele Amare, Sewbesew Yitayih, Kegnie Shitu, Demeke Demilew, Endalkachew Dellie, Andualem Yalew Aschalew, Biruk Fanta, Netsanet Worku, Ermias Solomon Yalew, Yohannes Abich, Getachew Azeze, Chanyalew Worku, Alemu Kassaw Kibret, Tsegaye G/Medhin, Melkamu Tamir Hunegnaw, Endalamaw Salelew, Goshu Nenko, Hailab Fekadu, Ayenew Molla

**Affiliations:** 1 Department of Human Nutrition, Institute of Public Health, College of Medicine and Health Sciences, University of Gondar, Gondar, Ethiopia; 2 Department of Epidemiology and Biostatistics, Institute of Public Health, College of Medicine and Health Sciences, University of Gondar, Gondar, Ethiopia; 3 Department of Health system and Policy, Institute of Public Health, College of Medicine and Health Sciences, University of Gondar, Gondar, Ethiopia; 4 Department of Environmental and Occupational Health and Safety, Institute of Public Health, College of Medicine and Health Sciences, University of Gondar, Gondar, Ethiopia; 5 Department of Health Promotion and Health Behavior, Institute of Public Health, College of Medicine and Health Sciences, University of Gondar, Gondar, Ethiopia; 6 Department of Occupational Therapy, School of Medicine, College of Medicine and Health Sciences, University of Gondar, Gondar, Ethiopia; 7 Department of Physiatry, School of Medicine, College of Medicine and Health Sciences, University of Gondar, Gondar, Ethiopia; 8 Department of Physiotherapy, School of Medicine, College of Medicine and Health Sciences, University of Gondar, Gondar, Ethiopia; 9 Department of Medical Nursing, School of Nursing, College of Medicine and Health Sciences, University of Gondar, Gondar, Ethiopia

**Correction: BMC Nutr 9, 102 (2023)**

10.1186/s40795-023-00758-1.

Following publication of the original article [1], the authors identified an error in the author name of Ermias Solomon Yalew.

The incorrect author name is: Ermiyas Solomon Yallew

The correct author name is: Ermias Solomon Yalew

The author group has been updated above and the original article [1] has been corrected.
